# Antimicrobial resistance profiles and mortality rates in intensive care unit patients having central line associated blood stream infection: a temporal analysis

**DOI:** 10.3205/dgkh000633

**Published:** 2026-03-02

**Authors:** Gargee Anand, Rijhul Lahariya, Asim Sarfraz, Bhaskar Thakuria, Prathyusha Kokkayil, Binod Kumar Pati

**Affiliations:** 1 All India Institute of Medical Sciences, Patna, Bihar, India

**Keywords:** antimicrobial resistance, central-line associated bloodstream infection, multidrug-resistant organisms

## Abstract

**Background:**

Patients with indwelling devices in the intensive care unit (ICU) face a heightened risk of infections, particularly central-line associated blood stream infection (CLABSI). With the aim of enhancing ICU prevention strategies, this study assessed CLABSI incidence, key pathogens, and their resistence profiles.

**Method:**

The retrospective study analyzed patients with central line catheter (CLC) from 2021 to 2023. Patients’ demographic details, CLC site, ICU stay, and blood culture reports including resistance were analyzed.

**Results:**

Among 3,761 patients, CLABSI incidence rates were 6.18 (n=18, 2021), 1.69 (n=15, 2022), and 3.75 (n=42, 2023) per 1,000 CL days. The predominant GNB included *Klebsiella pneumoniae* (25.3%), *Acinetobacter baumannii* (16%), and *Pseudomonas aeruginosa* (9.33%), while GPC included *Enterococcus species* (5.33%) and *Staphylococcus aureus* (2.66%). *Klebsiella (K.) pneumoniae* and *Pseudomonas (P.) aeruginosa* exhibited rising resistance to cephalosporins, carbapenems, BL-BLIs, and monobactams. *Acinetobacter (A.) baumannii* showed decreased resistance to carbapenems and BL-BLIs. *Staphylococcus (S.) aureus* exhibited decreased resistance to macrolides and lincosamides, while *Enterococcus* species showed increased resistance to macrolides and penicillin. *Candida* species (21.3%) were isolated in 2022, with one case of *Candida auris* in 2023. Multidrug-resistant (MDR) CLABSIs had statistically significantly higher mortality (p=0.042).

**Discussion:**

GNB showed increased resistance to cephalosporins, carbapenems, and BL-BLIs. In contrast, *S. aureus* demonstrated decreased resistance to lincosamides, while *Enterococcus* showed rising resistance to macrolides and penicillin, raising concern about treatment.

**Conclusion:**

These findings emphasize the urgent need for targeted stewardship against CLABSI.

## Zusammenfassung

**Hintergrund:**

Patienten mit Devices auf der Intensivstation sind einem erhöhten Infektionsrisiko ausgesetzt, insbesondere ZVK-assoziierten Blutstrominfektionen (CLABSI). Die Studie untersucht die CLABSI-Inzidenz, wichtige Krankheitserreger und deren Resistenzprofile, um Präventionsstrategien zu verbessern.

**Methode:**

In einer retrospektiven Studie wurden Patienten mit einem ZVK in den Jahren 2021 bis 2023 analysiert. Die demografischen Daten der Patienten, die Lokalisation des ZVK, die Aufenthaltsdauer auf der Intensivstation und Ergebnisse der Blutkulturen einschließlich Resistenz wurden analysiert.

**Ergebnis:**

Bei 3.761 Patienten betrug die CLABSI-Inzidenz 6,18 (n=18, 2021), 1,69 (n=15, 2022) und 3,75 (n=42, 2023) pro 1.000 ZVK-Tage. Zu den vorherrschenden GNB gehörten *Klebsiella (K.) pneumoniae* (25,3%), *Acinetobacter (A.) baumannii* (16%) und *Pseudomonas (P.) aeruginosa* (9,33%), während zu den GPC Enterococcus-Arten (5,33%) und *Staphylococcus (S.) aureus* (2,66%) gehörten. *K. pneumoniae* und* P. aeruginosa *zeigten eine zunehmende Resistenz gegen Cephalosporine, Carbapeneme, BL-BLIs und Monobactame. Acinetobacter zeigte eine abnehmende Resistenz gegen Carbapeneme und BL-BLIs. *S. aureus* zeigte eine abnehmende Resistenz gegen Makrolide und Lincosamide, während Enterococcus-Arten eine zunehmende Resistenz gegen Makrolide und Penicillin zeigten. Candida-Arten (21,3%) wurden im Jahr 2022 isoliert, mit einem Fall von *Candida auris* im Jahr 2023. Bei Nachweis multiresistenter (MDR) Erreger war Mortalität signifikant höher (p=0,042).

## Introduction

Healthcare-associated infections (HAIs) represent one of the most pressing global health challenges, affecting up to 30% of intensive care unit (ICU) patients and contributing significantly to morbidity and mortality, particularly in developing nations where the prevalence of HAIs is higher [1]. The burden of HAIs in the ICU setting is disproportionately driven by invasive devices, with vascular-access devices serving as the predominant source of infection in most cases [2]. Central line-associated bloodstream infections (CLABSIs) remain a major contributor to this burden, carrying the highest attributable mortality among all HAIs [1]. The pathogenesis of CLABSI is intricately linked to the use of central venous catheters (CVCs), where bacterial pathogens adhere to catheter surfaces and produce exo-polymeric biofilms. These biofilms confer antimicrobial resistance (AMR) by impeding drug penetration, promoting horizontal gene transfer, inducing persister states, and activating efflux systems, thereby rendering standard therapies less effective [3], [4], [5]. Terminally ill patients and those requiring prolonged CVC utilization are at particularly high risk, as biofilm formation not only shields bacteria from immune clearance but also fosters resistant phenotypes, complicating eradication and prolonging infection [3], [6]. Moreover, therapeutic challenges are compounded by delays in catheter removal—often unavoidable in critically ill patients—despite evidence that early removal (within 48 hours) improves microbiological cure and survival [7]. Treatment barriers are further intensified by the declining susceptibility of key Gram-negative pathogens, such as *Klebsiella (K.) pneumoniae* and *Acinetobacter (A.) baumannii*, to “Access” and “Watch” antibiotics, which increasingly necessitates reliance on last-resort agents [1]. In low- and middle-income countries (LMICs), these challenges are magnified by resource constraints, including the high cost of reserve antibiotics, limited laboratory capacity, and inadequate infection control infrastructure [8]. Emerging reports, particularly from Asia, highlight multidrug-resistant (MDR) Gram-negative organisms as the predominant CLABSI pathogens, thereby exacerbating AMR, complicating empiric therapy, narrowing treatment options, and contributing to poorer clinical outcomes [9], [10]. Given this context, CLABSIs represent not only a preventable but also a disproportionately fatal nosocomial infection, underscoring the urgent need for robust infection prevention practices, rational antibiotic use, and targeted surveillance in ICU settings [11]. Accordingly, the present study was designed to determine the incidence of CLABSI, assess the AMR profile and temporal trends of the causative pathogens over a three-year period, characterize the predominant microbial agents, and analyze the associated mortality rates among ICU patients in a tertiary care hospital in North-eastern India.

## Materials and methods

### Study design

This retrospective observational study was conducted in the adult and pediatric ICUs of a tertiary care center over a three-year period from 2021 to 2023. The study aimed to evaluate the incidence, microbiological profile and AMR trend of CLABSI-causing isolates among ICU patients and associated mortality.

### Patient recruitment

All patients who had a CVC in place for more than two calendar days were considered eligible for inclusion. Patients presenting with clinical features suggestive of bloodstream infection (BSI) underwent blood culture sampling for microbiological assessment to confirm or rule out bacteremia or fungemia. Those with secondary BSI were excluded from the analysis. After applying the inclusion and exclusion criteria, the final study population comprised 3,761 patients who required hospitalization during the study period.

### Data collection

Retrospective data were meticulously extracted from two institutional databases: the Hospital Information System (HIS) and the Hospital Infection Control surveillance forms. The HIS provided laboratory data, including blood culture results and antimicrobial susceptibility profiles, while surveillance forms supplied additional patient information such as demographics, diagnosis, central line site, ICU length of stay, date of discharge, in-hospital mortality, and daily bedside assessments for clinical signs suggestive of catheter-related infections. Patient and public involvement was not included, as the study exclusively relied on previously recorded departmental data.

Case definitions for CLABSI [12] and MDR organisms were adopted from established surveillance guidelines [13]. The CLABSI rate per 1,000 central line days was calculated by dividing the number of CLABSI episodes by the total number of central line days and multiplying by 1,000 [14].

### Statistical analysis

All data were entered into Microsoft Excel 2019. The normality of continuous variables was evaluated using Q-Q plots, histogram plots, and the Shapiro-Wilk test. Continuous variables were reported as mean ± standard deviation (SD) or median with interquartile range (IQR), depending on their distribution, while categorical variables were expressed as percentages or proportions. Bivariate comparisons of categorical variables were performed using the Chi-squared test or Fisher’s exact test, as appropriate. Trends in antimicrobial resistance were visualized using Python 3.10.12. Statistical analyses were conducted using SPSS version 22, and p<0.05 was considered statistically significant.

## Results

### Patient characteristics and CLABSI incidence

During the three-year study period from 2021 to 2023, a total of 3,761 ICU admissions met the inclusion criteria, accounting for 22,957 central line days. Among this study population, 75 participants developed CLABSI as defined by the Laboratory confirmed Bloodstream Infection 1 (LCBI 1) criteria, resulting in an overall incidence rate of 3.26 per 1,000 catheter days over the study period. The median age of affected patients was 38 years, and males accounted for 55 (73.33%) of cases. Table 1 (Tab. 1) depicts the factors associated with CLABSI.

### Temporal trends in CLABSI rates

Over the study duration, CLABSI rates varied, initially measuring 6.18 per 1,000 catheter days in 2021, decreasing to 1.69 in 2022, and rising again to 3.75 per 1,000 catheter days in 2023. This variability could indicate inconsistent implementation of infection control interventions such as hand hygiene, use of maximal sterile barriers during catheter insertion, and adherence to catheter care bundles among transitioning healthcare personnel. 

### Catheter site and CLABSI risk

A higher CLABSI incidence was noted with femoral catheterization at 4.29 per 1,000 catheter days, compared with internal jugular and subclavian access at 3.17 and 2.94 per 1,000 catheter days, respectively, indicating a significantly heightened risk with femoral catheterization (p=0.001).

### Microbiological profile of CLABSI

Gram-negative pathogens predominated amongst the microbiological etiological factors of CLABSI, accounting for 70.7% of isolates, with Gram-positive organisms comprising 8%. An emerging trend of *Candida* species was identified in 2023, accounting for 21.33%, of which non-albicans *Candida* comprised 14.66% of all CLABSI isolates.

Among Gram-negative pathogens, *K. pneumoniae* was the most frequently isolated pathogen, implicated in 25.3% of CLABSIs, followed by *A. baumannii* as the next predominant organism, accounting for 16% of microbiologically-confirmed CLABSI cases. Detailed microbial isolates are presented in Table 2.

Additional advent of MDR, intractable hospital outbreak causing fungal pathogen, *C. auris* documented its first incidence within the ICU in 2023, accounting for 1.3% of CLABSI. Additionally, in 2023, *C. indologenes* comprised 1.3% of CLABSI, reflecting its increasing recognition as an opportunistic pathogen within immunocompromised hospitalized populations.(Tab. 2)

### Antimicrobial resistance trends

Among *K. pneumoniae* isolates, increasing resistance was observed from 2021 to 2023 for aminoglycosides (66. 7% to 87.5%), cephalosporins (66. 7% to 97.5%), carbapenems (50% to 87.5%), fluoroquinolones (66. 7% to 100%) and beta lactams-beta lactamase inhibitors (BL-BLIs) (66. 7% to 87.5%), while resistance was decreased for folate pathway antagonists (from 83.3% to 75%). No resistance for colistin was documented in our study.

In terms of *A. baumannii*, decreasing resistance was seen from 2022 to 2023 for carbapenems, BL-BLIs and fluoroquinolones (100% to 83.3% each), and aminoglycoside resistance dropped from 83.3% to 75%, while resistance remained stable for cephalosporins (100%) and folate-pathway antagonists (66. 7%). Colistin was the sole agent demonstrating 100% susceptibility.

*P. aeruginosa* isolates exhibited 100% in-vitro resistance to all routinely used antimicrobial classes in 2023, with resistance rising over the study duration from 2021 to 2023 for cephalosporins, monobactams, fluoroquinolones and BL-BLIs (66.7% to 100%), while for carbapenems, resistance increased from 33.3% in 2021 to 100% in 2023. However, colistin retained its activity, with 100% susceptibility demonstrated in all *P. aeruginosa* strains, underscoring its continuing efficacy as one of the last-resort treatment alternatives for extensively-resistant strains of this high-risk nosocomial pathogen.

*Enterococcus* isolates exhibited escalating rates of in-vitro resistance to few antimicrobial agents over the study duration (penicillin and macrolides from 50% to 100%). However, complete retention of susceptibility for glycopeptides and oxazolidinones was observed across all enterococcal strains. Fluoroquinolone was 100% resistant from the beginning of study. This highlights the critical need for antimicrobial stewardship programs that promote judicious use of glycopeptides and oxazolidinones to ensure these last-resort drugs remain active against MDR *Enterococcus* strains circulating in the hospital.

**Figure 1:**
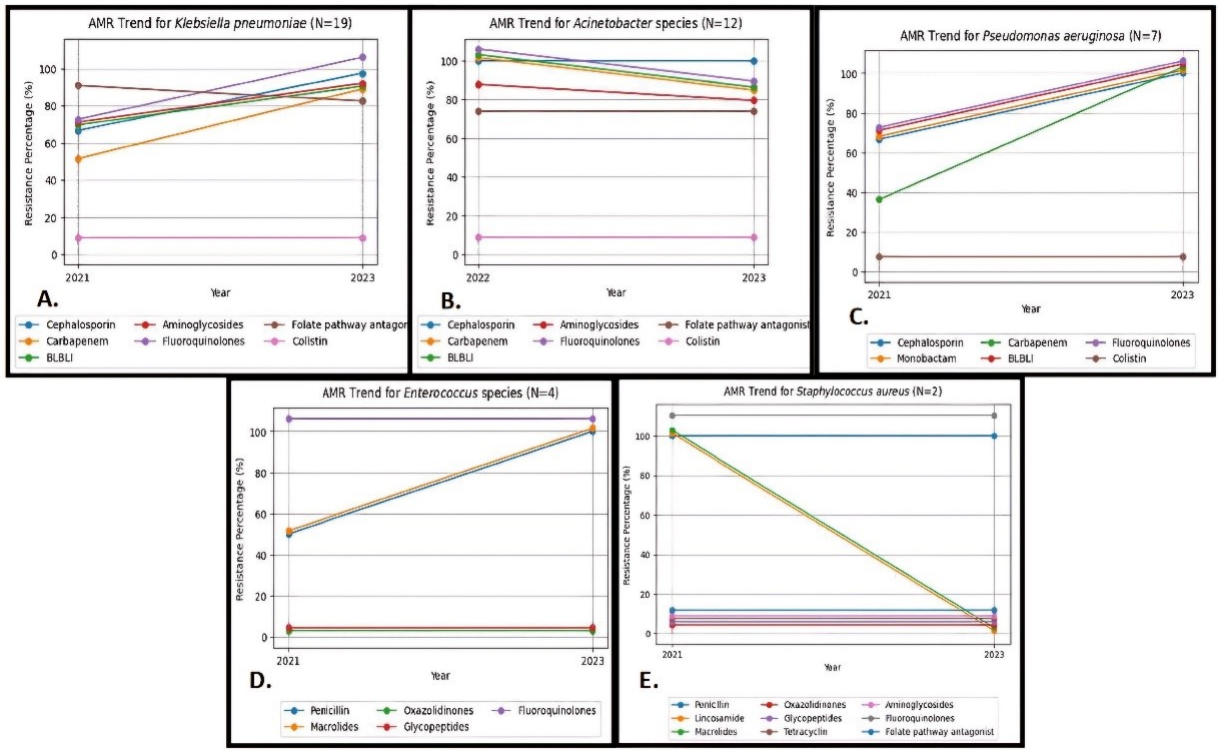
Antimicrobial resistance trend for A) *K. pneumoniae*, B) *A. baumannii*, C) *P. aeruginosa*, D) *Enterococcus* species, and E) *S. aureus*

Among *S. aureus* isolates, methicillin resistance was observed for all, concurrent with fluoroquinolone reaching 100% resistance from 2021 to 2023. However, sustained in-vitro susceptibility profiles (100%) for glycopeptides, oxazolidinones, folate pathway antagonists, aminoglycosides, and tetracyclines were maintained over the 3-year span, in contrast to decreasing resistance rate for lincosamide and macrolides (100% to 0%). The trend of AMR against these organisms is shown in Figure 1 (Fig. 1).

Furthermore, MDR bacterial strains comprised 9 out of 18 (50%) isolates in 2021, 13 out of 15 (86.6%) isolates in 2022, and 26 out of 42 (61.9%) isolates in 2023. Interestingly, the present analysis found a substantial increase in the proportion of MDR pathogens over the study duration, with a statistically significant rise from 50% (9/18) in 2021 to 86.6% (13/15) in 2022 (p=0.03). However, the difference in MDR incidence between 2022 (86.6%; 13/15) and 2023 (61.9%; 26/42) was not statistically significant (p=0.12).

### Mortality attributed to CLABSI

For the subset of 75 individuals with microbiologically confirmed CLABSI, the attributable mortality rate was 68% (51 deaths out of 75 cases). CLABSIs attributed to MDR bacteria were associated with a substantially elevated mortality rate (33 deaths vs 11 discharges) as compared to non-MDR bacteria (7 deaths vs 8 discharges) in the ICU patients, as evidenced by the statistically significant difference attained (p=0.04), implying that AMR nosocomial pathogen acquisition is an independent risk factor for greater mortality among critically ill patients with indwelling central catheters. In patients with fungal-associated CLABSIs, 5 were discharged, whereas 11 succumbed to the infection.

## Discussion

Among the 3761 critically ill patients meeting inclusion criteria for CLABSI, 75 cumulative episodes of central line-associated bacteraemia or fungemia were documented, with an incidence of 3.26 per 1,000 catheter days. This rate highlights the need for reinforced prevention efforts targeting modifiable risk factors for central line-related healthcare-acquired infection in this high-risk cohort. The CLABSI rate observed in the current study parallels the findings by Singhal et al. [15], Chudasma et al. [16] and Shin-Huei Kuo et al. [17] of 5, 3.69, and 3.47 per 1,000 catheter days, respectively, supporting the validity the incidence reported in this study.

The distribution of pathogens causing CLABSI can vary substantially across geographic settings and intensive care units. In previous studies, a preponderance of Gram-negative organisms was found in developing countries, while Gram-positive organisms were the major cause of CLABSI in developed countries [3], [18]. In the current investigation, Gram-negative organisms predominated, representing 70.7% of isolates, agreeing with findings by Kallel et al. [3] and Maqbool et al. [19], who similarly documented Gram-negative predilection. Conversely, a study by Ujesh et al. [20] found Gram-positive pathogens as the predominant etiological factor in their respective healthcare contexts. Also, among *Candida* species (21.3%), non-albicans *Candida* (68.8%) was found to be an emerging pathogen in our ICU setting, as compared to *C. albicans *(31.3%), which was in concordance with other studies [21]. These discordances reinforce that local antibiograms should highlight empiric antimicrobial selection, simultaneously with ongoing monitoring of changing microbial distributions underlying central line-associated sepsis within individual healthcare settings over time.

As also noted in previous literature, such as the study by Darji et al. [22], *K. pneumoniae* represented the predominant Gram-negative pathogen in our analysis of CLABSI cases. The high distribution of *K. pneumoniae* builds upon existing evidence which indicates that this organism is among the most frequent etiological factors of CLABSI globally, necessitating directed infection control efforts to curtail its circulation and suppress resultant infectious sequelae.

The present report elucidated 3-year trends pertaining to AMR profiles of pathogens implicated in CLABSIs. The most salient observation in this study was the increasing prevalence and MDR of *K. pneumoniae*, as opposed to the findings of Dickstein et al. [23]. Overall, MDR Gram-negative organisms exhibited declines during the study period, largely attributable to falling resistance rates to cephalosporins, fluoroquinolones, BL-BLIs, and aminoglycosides. With the exception of *K. pneumoniae*, *P. aeruginosa* and *Enterococcus* spp., MDR pathogens in the current study were generally found to be decreasing, largely due to the decreasing resistance towards cephalosporins, fluoroquinolones, and aminoglycosides. All the cases of *S. aureus* were methicillin resistant, similar to what Maqbool et al. reported [3].

Our findings demonstrated a significantly heightened CLABSI risk accompanying femoral CVCs compared to alternative vascular access sites, corroborating earlier evidence by Darji et al. [22]. The study by Al-Khawaja et al. [24] similarly determined femoral localization as an independent risk factor for device-associated infection and thrombosis. Moreover, the incidence of CLABSI with femoral catheterization is highest, but is lowest with subclavian access, which is in concordance with a different study [25]. The observed association between femoral catheterization and CLABSI occurrence may relate to the anatomical region and proximal microbial milieu, which differs considerably from the subclavian or jugular areas. Specifically, the anatomical location of the femoral vein is adjacent to the groin and perineum, meaning that its exposure to gut and genitourinary bacterial populations – including potential pathogens – is greater than in the upper chest and neck region [26], [27]. Additionally, factors such as moisture, poor hygiene, and hematoma risk can further increase infection likelihood [27]. Therefore, avoiding femoral access placement where viable alternatives exist could provide preventive benefits against CLABSI.

CLABSIs cause substantial attributable morbidity and mortality burdens. Within our population, a significant association was found between MDR-bacteria isolation and patient demise (p=0.04), with MDR CLABSI having a more than quadruple mortality risk compared to drug susceptible infections. These findings align with existing literature, such as the study by Mishra et al. [11], who similarly documented heightened attributable mortality accompanying CLABSI onset, especially involving drug-resistant pathogens. Consequently, antimicrobial stewardship and infection prevention strategies targeting highly resistant nosocomial pathogens may impart survival benefits to critically ill populations by suppressing deadly MDR central-line infections. This study offers crucial epidemiological and microbiological insights into CLABSI in critically ill patients, with strong relevance for antimicrobial resistance (AMR), infection prevention and control (IPC), and stewardship. The predominance of Gram-negative and non-*albicans Candida* spp., along with rising multidrug resistance of *K. pneumoniae*, underscores the need for dynamic, ICU-specific antibiograms to guide empirical therapy. The observed link between femoral catheterization and elevated CLABSI risk highlights a modifiable risk factor with immediate preventive implications. Future research should focus on prospective surveillance to track evolving resistance trends and assess the impact of targeted stewardship and catheter care bundles. This study’s novelty lies in integrating longitudinal resistance patterns with access-site-associated risk, offering actionable data to clinicians. It supports risk stratification, optimal line placement, and data-driven empiric antibiotic use, thereby enhancing clinical decision-making and strengthening the fight against HAIs in high-risk ICU settings.

## Limitations

The three-year duration and relatively small number of CLABSI cases limited the ability to fully characterize long-term antimicrobial resistance trends. With a larger sample size collected over a longer duration, the study could have provided more conclusive reference information on resistance patterns.

## Conclusion

In summary, this three-year analysis of ICU patients documented a CLABSI incidence of 3.26 per 1,000 catheter days, highlighting the need for reinforced preventive strategies targeting central line infection risks. Gram-negative pathogens, especially *K. pneumoniae*, predominated among isolated organisms. An escalating trend of MDR was observed in *K. pneumoniae*,* P. aeruginosa*, and *Enterococcus* spp. Femoral catheterization conferred a significantly higher CLABSI risk compared to subclavian and internal jugular access. Among Gram-negative pathogens implicated in CLABSI, maximal in-vitro susceptibility was seen for colistin (100%). All Gram-positive bacteria were sensitive to glycopeptides and oxazolidinones. When nothing else works against these nosocomial “superbugs”, careful use of these final-resort treatments can save lives. Options for eliminating MDR pathogens are very limited, and this rising resistance further reduces the choices of treatment. This urgently calls for antimicrobial stewardship initiatives in ICUs to conserve limited last-line antimicrobials against life-threatening MDR pathogens. 

The attributable mortality rate for microbiologically-confirmed CLABSI was 68%. These results highlight the critical need for multiple coordinated strategies, including infection prevention measures, antimicrobial stewardship, and careful central-line site selection to reduce deadly central-line related bloodstream infections in ICUs. Continued monitoring is necessary to understand emerging microbial and AMR risks, tailor appropriate initial antimicrobial choices, and measure the influence of infection control interventions on patient health improvement in the long run.

## Notes

### Authors’ ORCIDs

- Anand G: https://orcid.org/0009-0008-0473-389X
- Lahariya R: https://orcid.org/0009-0003-5769-4509
- Sarfraz A: https://orcid.org/0000-0002-6256-7649
- Thakuria B: https://orcid.org/0000-0001-6775-7639
- Kokkayil P: https://orcid.org/0000-0003-1114-7553
- Kumar Pati B: https://orcid.org/0000-0002-1948-2164

### Funding

None.

### Competing interests

The authors declare that they have no competing interests.

### Author Contributions

Gargee Anand, Rijhul Lahariya and Asim Sarfraz contributed to the study conception and design. Material preparation and data collection were performed by Gargee Anand, Rijhul Lahariya, Asim Sarfraz, Bhaskar Thakuria, Prathyusha Kokkayil and Binod Kumar Pati. Statistical analysis, interpretation and visualisation of data were done by Gargee Anand, Rijhul Lahariya and Asim Sarfraz. The first draft of the manuscript was written by Gargee Anand and Rijhul Lahariya. Gargee Anand and Rijhul Lahariya both contributed equally to the work. All authors commented on previous versions of the manuscript. All authors read and approved the final manuscript.

## Figures and Tables

**Table 1:**
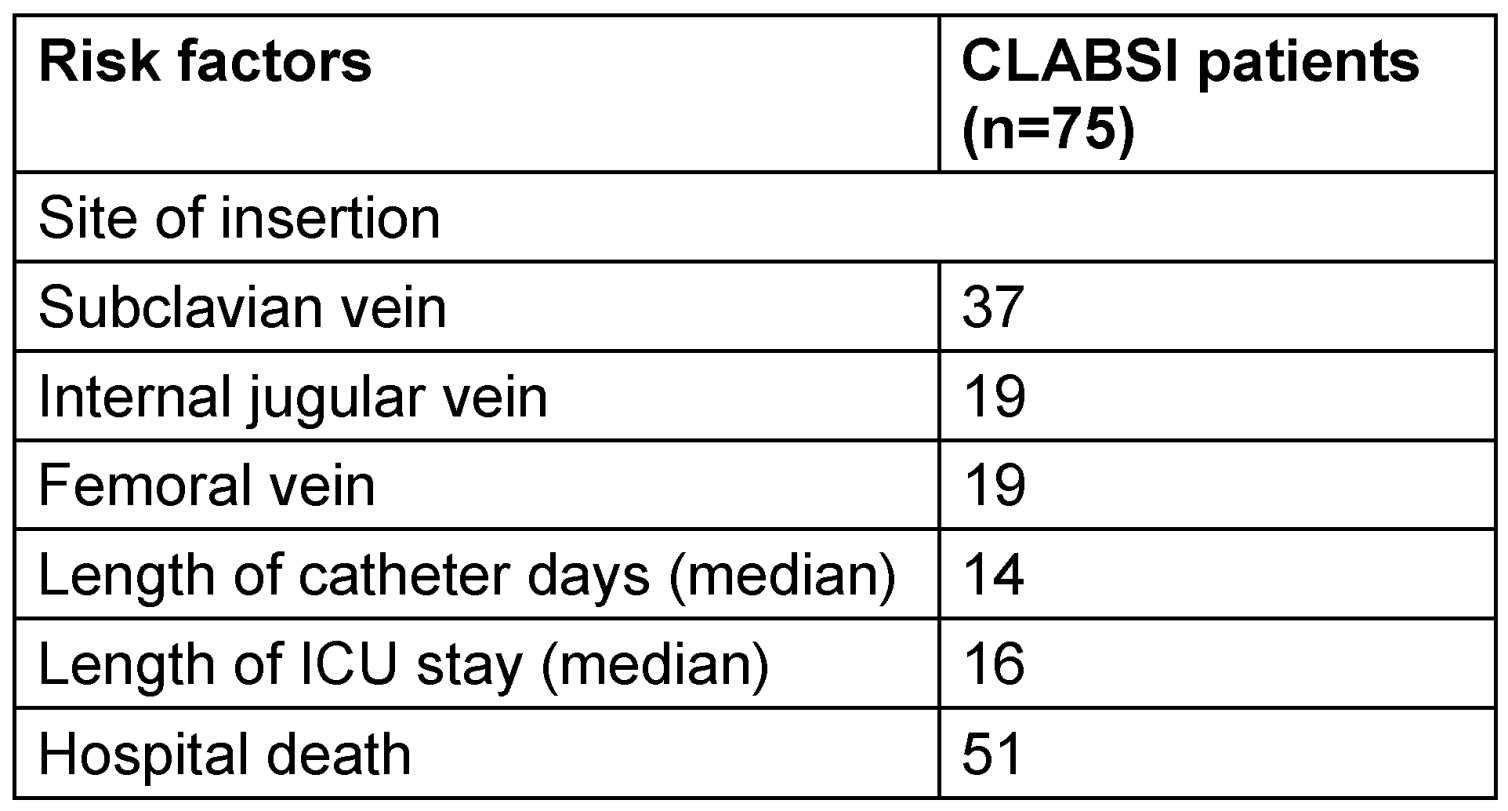
Risk factors associated with CLABSI

**Table 2:**
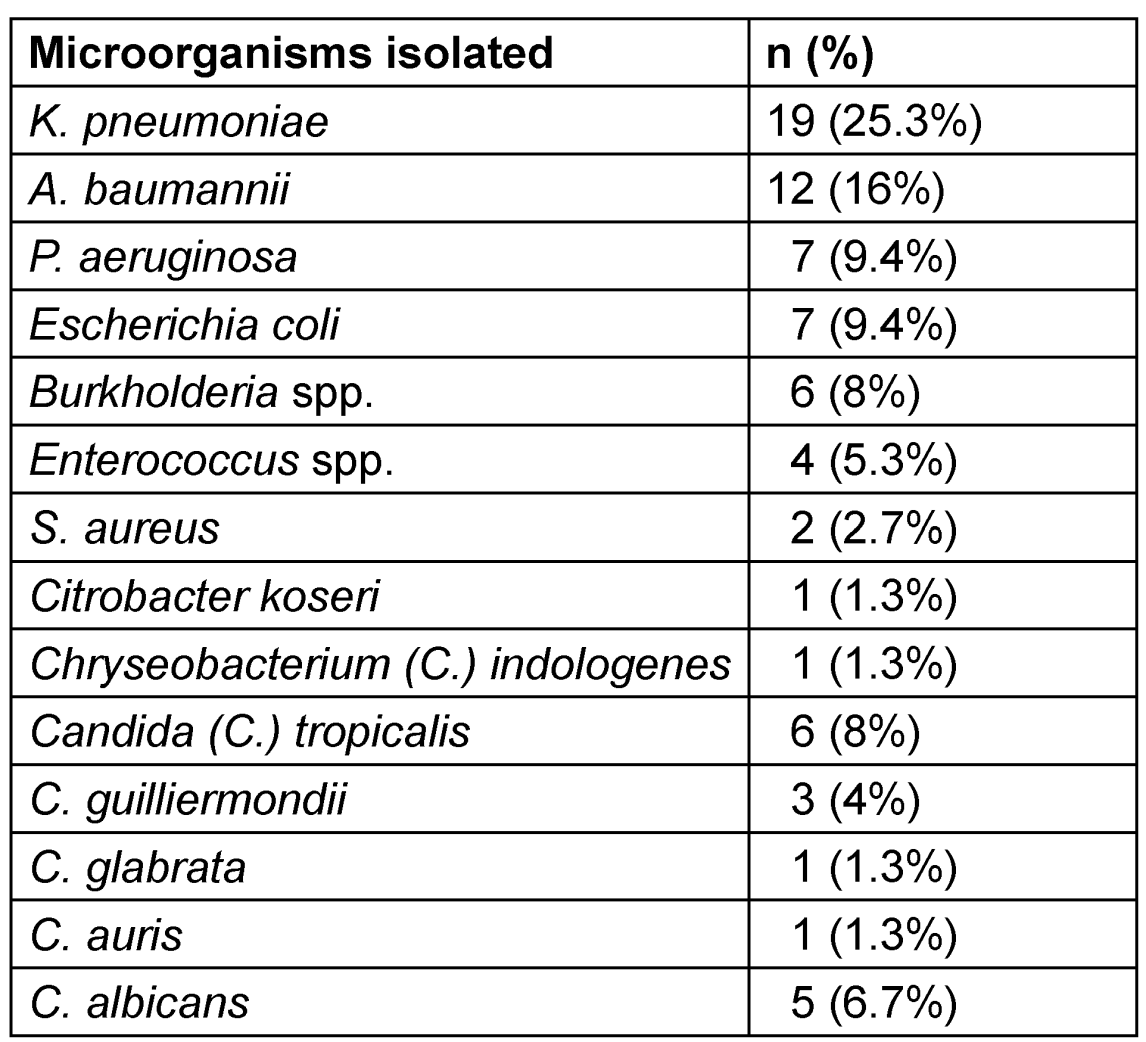
Microorganisms isolated from CLABSI patients
